# Are treatments for cervical precancerous lesions in less-developed countries safe enough to promote scaling-up of cervical screening programs? A systematic review

**DOI:** 10.1186/1472-6874-10-11

**Published:** 2010-04-01

**Authors:** Eric Chamot, Sibylle Kristensen, Jeffrey SA Stringer, Mulindi H Mwanahamuntu

**Affiliations:** 1 Department of Epidemiology, School of Public Health, University of Alabama at Birmingham, Birmingham, USA; 2 Centre for Infectious Disease Research in Zambia, 5977 Benakale Road, Northmead, Lusaka, Zambia; 3 Department of Gynecology and Obstetrics, University Teaching Hospital, Lusaka, Zambia

## Abstract

**Background:**

Since the mid-1990s, there have been growing efforts to prevent cervical cancer in less-developed countries through the development of innovative screening approaches such as visual inspection of the cervix associated with same day management of cervical lesions with cryotherapy or loop electrosurgical excision procedure (LEEP). In the past, promising cancer screening interventions have been widely promoted despite incomplete evidence, only to become the subject of intense controversies about ensuing net health benefit. Because the efficacy and effectiveness of the new protocols for global cervical cancer screening have not been well characterized yet, and as a contribution to the evaluation of the balance between the benefits and risks of these protocols, we reviewed the literature on the safety of cryotherapy and LEEP for cervical intraepithelial neoplasia (CIN) in low- and middle-income countries.

**Methods:**

We searched 12 databases (Medline, Google Scholar, Scopus, Cochrane Library, Web of Science, OCLC, PAIS International Database, WHO Global Health Library, CINAHL, Science.gov, NYAM Grey Literature Report, and POPLINE) for original research published between January 1995 and April 2009. Both peer-reviewed publications and items of "grey" literature were retrieved; no language restriction was applied. We calculated the median (minimum, maximum) reported rate for each harm considered. Because of limitations and heterogeneity in the data, no formal meta-analysis was performed.

**Results:**

The search identified 32 articles that reported safety data from 24 cryotherapy and LEEP studies. The combined sample consisted of 6,902 women treated by cryotherapy and 4,524 women treated by LEEP. Most studies were conducted in reference or research settings in Asia and Africa. Short-term harms of cryotherapy and LEEP appeared to be similar to those described in the literature from high-income countries. Information was sparse on HIV-related harms and long-term reproductive outcomes of treatment.

**Conclusions:**

When performed in resource-limited settings by qualified providers, cryotherapy and LEEP are not associated with excess harm. However, available data are insufficient to propose fully evidence-based protocols for routine screening of HIV-infected women and women of reproductive age.

## Background

In resource-limited countries where cervical cancer is typically the most common cause of premature death among middle-aged women, an estimated 230,000 women die each year from invasive cervical cancer (ICC) [1,2]. In many parts of the developing world, age-standardized incidence rates of ICC are ≥ 4-fold higher than in North America and Western Europe, reaching values in excess of 30-to-50 per 100,000 women in large areas of sub-Saharan Africa, Latin America, the Caribbean, South Asia and Oceania [1].

Whereas cervical cancer burden in industrialized countries decreased sharply after the widespread introduction of effective cytological screening programs, these favourable results have not been replicated in the developing world. In low-income countries resources and infrastructure proved insufficient to offer quality Pap smear screening to more than a small fraction of adult women and high rates of loss to follow-up associated with multi-visit screening protocols further hampered success. Hence, with evidence suggesting that human papillomavirus (HPV) vaccine will not substitute for secondary prevention [3], attention has been redirected toward the development of novel and simple cervical cancer screening approaches more suitable for use in resource-poor settings. These efforts have led to the emergence of screening of middle-aged women by means of visual inspection of the cervix (after application of acetic acid or Lugol's iodine), or high-risk HPV DNA testing, coupled with immediate or shortly deferred management of abnormalities, as potentially viable and cost-effective strategies to reduce ICC incidence in less-developed countries [4-6].

This encouraging advance should be viewed with the understanding that cancer screening has often been controversial. Much debate in particular has surrounded the tendency for the medical and public health community to aggressively promote new screening interventions at the population level before obtaining solid evidence that the benefits of early detection reasonably outweigh the harms [7-9]. According to the International Agency for Research on Cancer (IARC [10]), pilot cervical cancer screening programs are being implemented in more than 40 developing countries. Although research findings generally support the appropriateness of low-technology cervical cancer screening, important questions remain in particular in terms of screening test, definition of target populations, long-term quality control, and human resource commitment [11-14]. A major shortcoming of "screen-and-treat" approaches without tissue diagnosis is that up to 80%-90% of women who receive treatment following an abnormal primary screening result are free of CIN 2 or worse [15,16]. A substantial proportion of these women are at low risk for progression to high-grade lesion or ICC, and therefore do not truly benefit from therapy. Cumulatively, potential harms experienced by screening participants are not only detrimental in themselves; they could also negatively affect future screening coverage--a key determinant of program cost-effectiveness [6]. Finally, there are at least theoretical reasons to fear that minor adverse effects of treatment may evolve into severe complications more frequently in low- than in high-income countries, since women living in less affluent settings are more likely to be affected by comorbidities (e.g., HIV or anaemia) and to lack timely access to adequate medical care. For these reasons, a crucial precondition for the global scale-up of single-visit screening programs is the guarantee that acceptable safety levels are achieved under *routine *conditions of implementation.

Looking at pilot studies in low-resource settings, cryotherapy (i.e., freezing of cervical lesion by application of liquid nitric oxide or carbon dioxide [17]) performed by mid-level providers has been used for the management of up to 85%-90% of abnormalities detected by screening [18,19]. Typically, women ineligible for cryotherapy were referred for further evaluation and therapy by loop electrosurgical excision procedure (LEEP [20]). In 2003, the Alliance for Cervical Cancer Prevention (ACCP) reviewed evidence published in 1955-2001 on the safety of cryotherapy for the management of CIN [21]. Most of the studies included in the review were conducted in high-income countries and focused on treatments performed by high-level providers under colposcopic guidance. To complement this work, we report here on the results of a systematic review of the literature published between 1995 and 2009 on the safety of cryotherapy and LEEP for the management of cervical lesions of any grade in low- and middle-income countries.

## Methods

### Search strategy and selection criteria

We searched Medline (PubMed), Google Scholar, Scopus (Elsevier), the Cochrane Library, Web of Science, OCLC (Paper First and Proceedings First), PAIS International Database (EBSCO), WHO Global Health Library, CINAHL, Science.gov, the New York Academy of Medicine Grey Literature Report, and POPLINE for single reports, abstracts and other "grey literature" published in any language between January 1995 and April 2009. We chose 1995 for our starting point since the modern concept of low-technology single-visit screening for cervical cancer emerged around that time. With the support of a medical librarian, we combined more than 20 key search terms (Additional file 1: search algorithm in Medline) to identify all primary studies in which women with cervical intraepithelial neoplasia of any grade were treated with either cryotherapy or LEEP.

For the main search, two reviewers independently judged each publication for eligibility by reviewing the title and abstract. Discrepancies were resolved through discussion between reviewers. References were excluded at this stage if CIN therapy was mentioned only incidentally or if the research was conducted in a high-income country [22]. No restriction was imposed by study design. The references of the articles obtained from the initial search were manually screened for additional studies. We read the full text of each citation that reported original clinical data and appeared to include information on treatment by cryotherapy or LEEP.

### Data abstraction and quality assessment

During the main search, two reviewers independently used a pretested form to extract the following data: year of publication; country; study design; population, indications to treatment; treatment setting and characteristics; follow-up; and adverse outcomes. Disagreements among reviewers were arbitrated by a third investigator. Quality of safety reporting was based on the following four criteria: (1) recruitment completeness among members of a clearly-defined cohort; (2) report of explicit definitions that allow for reproducible ascertainment of two or more harms; (3) assessment of patients status within seven days to one month of treatment; and (4) attrition rate ≤ 20% until first follow-up assessment [21,23].

### Analysis

Because the methods and quality of harm ascertainment varied considerably from one study to the other, we only estimated the minimum, median, and maximum rates of each adverse outcome across studies.

## Results

**Figure 1:**
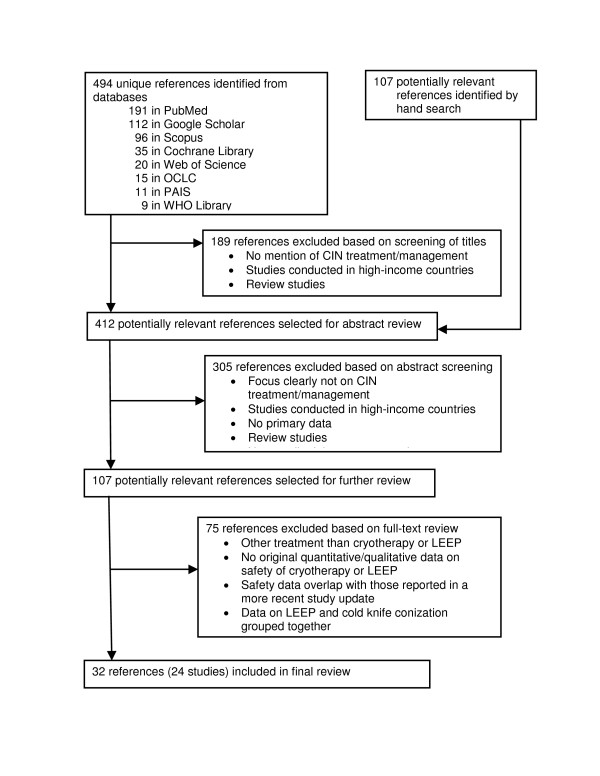
**Flowchart of the systematic search process**. OCLC = Online Computer Library Center; PAIS = Public Affairs Information Service International Database; CINAHL = Cumulative Index to Nursing and Allied Health Literature.

The literature search produced 32 articles (31 publications and 1 conference report; Figure 1) that reported on non-overlapping harms of cryotherapy, LEEP, or both in 24 studies fulfilling the inclusion criteria (Additional files 2 and 3). For five studies, non-overlapping safety data were found in more than one article.

### Study characteristics

Eight studies provided data on harms of cryotherapy [24,(25,26),27-32], 13 on harms of LEEP [19,33-47], and 3 on harms of both cryotherapy and LEEP [(48-50),(51,52),(53,54)] (Additional files 2 and 3).

Indication for treatment was a biopsy- or colposcopically-confirmed diagnosis of CIN in 17 studies (71%) and an abnormal screening test result without further diagnosis work-up ("screen-and-treat" approach) in 6 studies (25%); 1 study (4%) included women with either indication for treatment. Age of participants was reported for 17 of the 24 studies and ranged from 13 to 96 years (Additional files 2 and 3); most women were in their late 20s to early 50s. Eleven studies were from Asia, 7 from Africa, 4 from Latin America, and 2 from Eastern Europe. Five (21%) studies were randomized controlled trials, 12 (50%) were prospective cohort studies, and 7 (29%) were retrospective case series. Median sample size was 574 for cryotherapy and 149 for LEEP. In total, the review included evidence on 6,902 women who underwent cryotherapy and 4,524 women who underwent LEEP. Per our criteria, study quality for harm assessment was low: only 5 studies (21%) met 3 or more criteria (Additional files 2 and 3).

### Treatment

Contraindications to treatment were discussed in all of the cryotherapy studies and in 14 (88%) of the LEEP studies (Table 1). Community outreach was the prime method of enrollment for studies that focused on cryotherapy (64%). In LEEP studies, participants were typically recruited among outpatients of gynecology clinics or tertiary care centers (63%). Treatment was offered in fixed or mobile primary care centers in 8 (73%) of the cryotherapy studies, and in gynecology clinics or tertiary care centers in 12 (75%) of the LEEP studies (missing information, 1 study). LEEP was virtually always performed by a doctor in a single health center whereas cryotherapy was commonly performed by mid-level care providers (primarily nurses) located in more than one health facility. Information on number of operators was inconsistently reported, although cryotherapy appears to have been more frequently performed by multiple operators than LEEP. As expected, local anesthesia (general anesthesia in [44]) was used to control pain during LEEP, but not during cryotherapy. Information on prophylactic antibiotic therapy was inconsistently reported; routine antibiotic therapy was described in 4 (36%) cryotherapy studies and 3 (19%) LEEP studies.

**Table 1:** Summary information about treatment and treatment delivery system

	Cryotherapy*		LEEP*	
	n	(%)	n	(%)
Indications/contraindications**				
Restrictions based on lesion size and extension	8	(72.7)	0	(0.0)
Transformation zone entirely visible	6	(54.5)	2	(12.5)
No evidence of invasive disease	6	(54.5)	5	(31.3)
No history of screening or treatment for CIN	7	(63.6)	2	(12.5)
No gynecologic abnormality^†^	4	(36.4)	1	(6.3)
No severe cervicitis	1	(9.1)	2	(12.5)
Restrictions for pregnant women^††^	6	(54.5)	3	(18.8)
Contraindication to cryotherapy	0	(0.0)	5	(31.3)
Enrollment**				
Community outreach	7	(63.6)	4	(25.0)
Primary care/local health centers	3	(27.3)	1	(6.3)
Gynecologic clinic/tertiary care	3	(27.3)	10	(62.5)
Not reported	0	(0.0)	1	(6.3)
Type of treatment center**				
Gynecologic clinic/tertiary care	4	(36.4)	12	(75.0)
District hospital	2	(18.2)	2	(12.5)
Primary care center/mobile unit	8	(72.7)	1	(6.3)
Not reported	1	(9.1)	1	(6.3)
Number of treatment centers				
One	5	(45.5)	16	(100)
Two or more	5	(45.5)	0	(0)
Not reported	1	(9.1)	0	(0)
Operator				
Gynecologist, surgeon	1	(9.1)	4	(25.0)
Doctor, general practitioner	2	(18.2)	4	(25.0)
Nurse, "less qualified personnel"	5	(45.5)	1	(6.3)
Not reported	3	(27.3)	7	(43.8)
Number of operators				
One	1	(9.1)	1	(6.3)
Two to five	1	(9.1)	5	(31.3)
Six or more	5	(45.5)	0	(0)
Not reported	4	(36.4)	10	(62.5)
Control of pain				
No specific measure/Non-narcotic analgesics as needed	5	(45.5)	0	(0.0)
Local anesthesia	0	(0.0)	12	(75.0)
General anesthesia	0	(0.0)	1	(6.3)
Not reported	6	(54.5)	3	(18.8)
Antibiotherapy (pre-treatment)				
Signs of cervicitis or STD	3	(27.3)	2	(12.5)
All women	4	(36.4)	3	(18.8)
Not reported	4	(36.4)	11	(68.8)

*Cryotherapy, 11 studies; LEEP, 16 studies.

**Total percent add to more than 100%, because some studies are represented in more than one item category.

^†^Abnormalities such as polyp or cervical atrophy, for instance.

^††^Different gestational ages across studies.

### Technical factors potentially influencing safety

Ten cryotherapy studies reported that a double-freeze technique had been used in all, or most, of the patients [24,25,27-31,48,51,54]; 6 specified the nature of the refrigerant employed (nitric oxide, 4 [24,29,48,51]; carbon dioxide, 2 [25,31]); and 7 reported information on the type [24,27,30,48,51,54] and the size [24,25,27,30,48,51] of the cryoprobe selected. For LEEP, loop size was adapted to the size of the lesion in 8 studies [33,37,39-41,44,47,52] (fixed 20 mm loop-size [46]; unreported size [19,38,42,43,45,49,52]). In 7 studies, cone depth was ≥ 10 mm in most patients [33,37,39,40,44,45,47] (unreported depth [19,41-43,46,52,54]). Excision was performed with blended current in 5 studies [33,37,41,44,47] and in cutting mode in 2 studies [45,54]; (mode unreported [19,38-40,42,43,47,49,52]). Finally, fulguration of the wound (± application of Monsel's solution) to provide hemostatis was undertaken routinely in 10 studies [33,37-40,44-46,51,54] and as needed in 2 [41,47] (unreported [19,42,43,52]).

### Pain

Information on pain and cramping was provided in 10 cryotherapy studies [24,26,27,29-32,48,51,54] and 4 LEEP studies [35,38,49,54] (Additional file 4). It was generally unclear whether the patients' or the providers' perceptions were recorded. Mild-to-moderate pain during or immediately after treatment was reported by nearly one third of women who underwent cryotherapy and by less than 5% of women who underwent LEEP (severe pain: 1%-4%). Longer lasting pain, or pain associated with the next menses, was common and documented considerably more often after LEEP (median, 67%; mean duration, 1-to 2-days [32]) than after cryotherapy (median, 9%).

### Vaginal discharge

Nine cryotherapy studies [24,26,29-32,48,51,54] and 5 LEEP studies [35,38,43,49,54] assessed the occurrence of vaginal discharge post-treatment. Perhaps reflecting the lack of standard method of assessment for mild side effects, rates of *watery *discharge after cryotherapy varied considerably across studies (9% to 92%; median, 65%) compared to rates of *offensive *or *disconcerting *discharge (1% to 11%, except in [54]; Additional file 4). Virtually all women (79% to 100%) who underwent LEEP noted some form of vaginal discharge. Rarely evaluated, duration of discharge ranged from one to three weeks in one cryotherapy study [31] and averaged 14 ± 4.6 days in one LEEP study [35]. No study attempted to measure the amount of discharge.

### Bleeding

Bleeding was an adverse outcome reported in all the studies reviewed, except two [28,31]. Among women treated with cryotherapy, *intraoperative *or *immediate postoperative *bleeding was uncommon (<2%; Additional file 4); *slight delayed *bleeding occurred in up to 10% to 40% of women, in particular in association with menses; and hemorrhage requiring hospitalization or transfusion was documented in only 1 study (1 of 949 women; 0.1% [29]). In contrast, bleeding was frequent and sometimes severe among women treated with LEEP. Typically, *uncomplicated post-LEEP *bleeding was experienced by all women for 3-to-4 days on average. One LEEP study reported that *clinically significant early *bleeding occurred in 2% of women [54]; whereas others indicated that application of Monsel's solution or electrocautery were needed to control hemorrhage in up to 3.3% of women during the first 24 hours post-treatment [19,33,40] and in 1.5% to 5.2% of women during the following days and weeks [33,37,40,41,45,46,49]. Three studies reported that suture to control bleeding or transfusion had been necessary in up to 0.8% of women [33,38,52]. Finally, 2 studies mentioned that hysterectomy had been required to control postoperative bleeding in some patients. In a preliminary report of the Osmanabad screening project in India [50], Sankaranarayanan and coll. indicated that hysterectomy was performed to manage hemorrhage in 1 woman out of the first 50 treated by LEEP whereas no other instance of emergency hysterectomy was recorded in the next 1090 women who received LEEP through the program (overall rate, 0.09% [49]). In a Chinese series of patients who underwent hysterectomy within 6 months of LEEP for CIN, hysterectomy was motivated by postoperative hemorrhage of over 500 ml in 2 women out of 73. Assuming that 5 percent of all the women treated by LEEP had a hysterectomy as a definitive cure for CIN (information not provided), 0.14% of all LEEP treatments were followed by an emergency hysterectomy.

### Infection

Infectious complications were discussed in 7 cryotherapy and 10 LEEP studies (Additional file 4). Cervical tenderness, fever, or wound infection requiring antibiotherapy were documented in 0.0% to 2.6% of women treated with cryotherapy [25,31,48,51,54] and 0.0% to 10.0% (median, 1.2%) of women treated with LEEP [19,33,38-40,44,46,47,49,54]. Pelvic inflammatory disease was rare after both cryotherapy (0.0% to 0.1%) [24,25,30,48,51] and LEEP (0.0% to 0.7%) [38,40,44,47,49].

### Other short-term adverse outcomes

Accidental thermic lesions of the vaginal wall occurred in 0.1% to 0.8% of women during cryotherapy (freezing) [28,48,51] and in 0.4% to 4.4% of women during LEEP (burning [38,49]; Additional file 4). Sidewall vaginal lacerations during LEEP was a reported cause of intraoperative bleeding requiring suture hemostasis in one study (0.3% of women) [19]. No bladder or bowel injuries were noted in any LEEP study, even though several accounts of such complications have been documented in high-income countries [55,56]. Minor spontaneously-resolving complications of local anesthesia were noted in 2 LEEP studies after submucosal infiltration of 2% lidocaine with 1:80,000 epinephrine (hypertension among 26% [47] - and uncontrollable trembling of the lower extremities among 0.3% of women [19]). In one cryotherapy study [29], 25% of women presented at their 1-month follow-up visit with a new troubling symptom requiring clinical evaluation (untreated control group, 10%). Unscheduled visits were documented in 4.4% to 9.7% of women in 3 cryotherapy studies [27,29,30] and in 5.6% of women in one LEEP study [43] (Additional file 4). Among women who had undergone cryotherapy, the visit led to the ambulatory treatment of a complication in 0.0% to 2.2% of women [27,30] and to hospital admission in 0.0% to 0.2% of women [24,29,30,48,51]. Five studies, 3 of cryotherpy [27,30,51] and 2 of LEEP [43,52], recorded no major short-term complication in any woman.

### Cervical stenosis and reproductive outcomes

Out of 6 cryotherapy studies [24,30,32,48,51,54] and 8 LEEP studies [19,33,38-40,47,49,54] (Additional file 4) that assessed risk of cervical stenosis (typically 6-12 months post-treatment), evidence that such complication occurred was reported in only 2 LEEP studies (3.3% and 8.0% of women) [40,47]. Despite the potentially severe consequences of cervical stenosis, in particular in premenopausal women, no case definition was provided in any of the reports. Instead, authors most commonly used descriptors such as "functional cervical stenosis", "clinically apparent stenosis", or "complaints consistent with cervical stenosis". We found no more than anecdotal data on risks associated with cryotherapy and LEEP performed during pregnancy and no data on long-term fertility and pregnancy outcomes.

### HIV infection

In a randomized trial [29], the 6-month risk of HIV seroconversion was similar among HIV-negative women who underwent and did not undergo cryotherapy at baseline (RR, 1.06; 95% CI, 0.59-1.53), but the authors noted that the study had only 80% power to detect a 2-fold increase in seroconversion risk. We did not find any other study that assessed whether women treated for CIN were at higher risk of acquiring or transmitting HIV infection. Although data were sparse, there was no evidence that harms of cryotherapy and LEEP were more common, or severe, in HIV-infected women compared to HIV-uninfected women [19,33,35,40,53].

### Comparisons betweens safety of cryotherapy and LEEP

Only one randomized controlled trial [53] made direct comparisons betweens adverse outcomes of cryotherapy and LEEP. Level of pain and frequency of moderate bleeding during treatment were similar among the 200 women allocated to the cryotherapy arm and the 200 women allocated to the LEEP arm. At two-week follow-up, however, compared to women treated with cryotherapy, women treated with LEEP were more likely to report secondary bleeding (79.0% versus 40.0%; p < 0.001) and offensive discharge (79.0% versus 68.2%; p = 0.03), and less likely to report watery discharge (78.5% versus 92.4%; p < 0.001).

### Risk stratification

Few studies attempted to identify risk factors for complications of cryotherapy or LEEP. The factors considered included pregnancy for cryotherapy [31], and age [35], histologic grade [35,36], lesion size [35], and number of treatments for LEEP [35,36]. None of the studies were sufficiently powered for this assessment, however, and only repeat LEEP (versus first LEEP) was found to be associated, in one study, with a marginally increased risk of complication (persistent bleeding) [34].

## Discussion

Our systematic review of the literature suggests that the short-term adverse outcomes of cryotherapy and LEEP for cervical abnormalities performed by highly skilled providers, or carefully-trained and well-supervised mid-level providers, in low- and middle income countries do not differ substantially from the outcomes previously documented for these procedures in resource-rich countries. Precise estimates of safety rates associated with cryotherapy and LEEP in less-developed countries cannot be determined at this point, however, since information remain sparse, and few of the reviewed studies, if any, met current standards for accurate and comprehensive reporting of harms [23,57,58].

Overall, the high degree of heterogeneity in quality, reporting, and study results complicated the tasks of abstracting and summarizing data. Most reports focused on a narrow array of perioperative and post-intervention outcomes, and few included any patient-centered measure, assessment of functional outcomes, or evaluation of long-term fertility and pregnancy outcomes. In a majority of reports the description of the data collection methods was superficial. Typically, the study personnel simply recorded the complications that they attributed to treatment; operational definitions of harms were not provided; and degree of harm was not reported on explicitly-defined severity scales. A minority of studies accounted for the possibility that patients experiencing complications may seek care at a non-study health institution. Finally, only one study reported information from a control group of untreated women to account for background risks [29].

### Assumptions about safety of cryotherapy in recent decision models

In recent years, several decision models have been proposed to compare the cost-effectiveness of a range of cervical cancer screening protocols in resource-limited settings [4-6]. In these analyses, minor short-term complications requiring a clinical visit and outpatient treatment were modelled to occur in 4% to 5% of women undergoing cryotherapy, and more severe complications requiring 1-2 days of hospitalization in 0.5% to 1% of women. Both our review and the review of the ACCP [21] suggest that these assumptions were likely to be conservative.

### LEEP safety

In LEEP outcome studies conducted in *resource-rich countries*, about 70% of women experienced vaginal discharge for a median of two weeks, typically with light bleeding during the first few days, and some 40% of women complained of menstrual-like pain during an average of three days [59]. Minor complications leading to patients returning to the clinic for evaluation occurred in less than 15% of women, and major short-term complications requiring hospitalization, surgery, or both occurred in no more than 2.0% of women [56,59,60]. Asymptomatic cervical stenosis (i.e., a stenosis revealed only by the inability to pass a fine probe through the cervical canal) was observed in up to 4%-6% of women [60,61]. Our findings appear to be of the same order of magnitude, although few of the studies included in our review reported on the frequency of complications leading to outpatient visit or hospitalization.

Although innovative models of referral and management services are being tested in resource-limited settings [19], it must be noted that LEEP is still typically performed by high-level providers in district or provincial facilities. Because the LEEP studies we reviewed were conducted by highly qualified and well-trained operators in teaching facilities, it is unclear whether the favourable results obtained thus far in less-developed countries will be maintained when screening programs are scaled up and LEEP is performed in remote areas by less skilled providers. More generally, it is difficult to see how the need for surgical providers will be met in the foreseeable future given the current scarcity of health resources and the shortage in health care workers in many part of the world.

### Literature gaps

The most substantial knowledge gap is that the safety of cryotherapy and LEEP has been the least studied in low-resource settings where the consequences of complications are potentially more serious. Experience with cryotherapy and LEEP, in women living with HIV/AIDS in particular, remains limited. In Africa, prevalence of CIN in these women reaches 50% to 76% [62,63]. Hence, most programs are likely to adopt aggressive screening protocols in HIV-infected women and to monitor HIV-infected women treated for cervical abnormalities every 6 months for persistent or recurrent lesions. Given the low efficacy of cryotherapy in women living with HIV/AIDS [64], there is a risk, in the absence of adequate quality assurance and performance feedback, that screening personnel engages in cycles of increasingly aggressive interventions that result in substantial cumulative harm without necessarily leading to meaningful long-term benefit to the patient.

Our systematic review identified only one study that assessed the risk of a woman becoming infected with HIV after cryotherapy; none that assessed risk of HIV infection after LEEP; and none that assessed the risk of HIV transmission after cryotherapy or LEEP from an infected woman to an uninfected partner. This is a major concern given what is known about the role of genital ulcerative diseases in the transmission of HIV, the ever-present risk of breeches in the process of instrument disinfection and sterilization in resource-deprived settings, and existing evidence that high levels of HIV-1 are shed in the vaginal secretions of infected women treated for CIN [65].

Finally, our review did not find any study that assessed pregnancy outcomes after first or repeated treatments of cervical abnormalities. This is troubling since HIV-infected women diagnosed with CIN in sub-Saharan Africa are generally of reproductive age and living in societies that allocate a central importance to fulfilling marital and reproductive goals. Although evidence about the effects of cryotherapy and LEEP on future pregnancies in high-income countries have been negative or equivocal [58], the medical literature suggests both that cervical length at 24-28 weeks of gestation is inversely related to the incidence of preterm birth [66] and that cervical length measured by transvaginal ultrasonography during pregnancy is reduced after both cryotherapy and LEEP [67]. In a recent systematic review, pooled risk of preterm delivery was 2.6 times higher when depth of resection after LEEP was ≥ 10 mm versus <10 mm [68]. Despite conflicting results, a history of LEEP has also been found associated in some studies with increases in risk of preterm birth (<34 - 37 weeks' gestation), premature rupture of the membranes, and low birth weight infants (<2500 g) [58,68,69]. Large studies are needed to identify the conditions under which cryotherapy and LEEP can be safely performed in women of reproductive age living in less-developed countries.

### Strength and limitations

Our systematic review of recent studies of the harms of cryotherapy and LEEP in developing countries includes only one study [32] that was discussed in the earlier report by the ACCP [21]. Our findings should be interpreted with caution since source data had substantial limitations. It appears likely that unpublished studies were performed with fewer resources and under lower standards of quality assurance. Hence, even though negative findings may have remained unpublished, there is also a possibility that results presented in our review underestimate the true adverse outcomes of cryotherapy and LEEP performed under routine conditions in peripheral, resource-deprived, settings.

## Conclusions

Our review does not provide evidence that cryotherapy and LEEP for cervical lesions in low- and middle-income countries are associated with more frequent, or more severe, adverse outcomes than in resource-rich contexts. Our review does indicate, however, that current data are insufficient to fully inform decisions on protocols for cervical cancer screening in HIV-infected women and women of reproductive age. Safety of CIN therapy should be further assessed in these populations under conditions of routine delivery by lower-skilled providers before screening becomes widely offered outside of demonstration programs. Finally, alternatives to cryotherapy and LEEP are needed. Since recent developments in the understanding of the HPV life cycle offer new prospect for identifying targets for drug design [70,71], research on non-surgical therapies for CIN should be encouraged.

## Competing interests

The authors declare that they have no competing interests.

## Authors' contributions

EC, SK, JS and MM jointly formulated the research question, defined the inclusion/exclusion criteria, interpreted the findings, and wrote the manuscript. EC and CF (see Acknowledgements) served as primary reviewers. SK served as third referee.

## Pre-publication history

The pre-publication history for this paper can be accessed here:

http://www.biomedcentral.com/1472-6874/10/11/prepub

## Supplementary Material

Additional file 1 Search algorithm in Medline. Click here for file

Additional file 2 Characteristics of cryotherapy studies, and studies of cryotherapy versus LEEP, included in the review. Click here for file

Additional file 3 Characteristics of LEEP-only studies included in the review. Click here for file

Additional file 4 Summary of evidence on safety of cryotherapy and LEEP for CIN in less-developed countries, 1995-2009. Click here for file
