# Supplementary material for: Are treatments for cervical precancerous lesions in less-developed countries safe enough to promote scaling-up of cervical screening programs? A systematic review
Source: BMC Womens Health. 2010 Apr 1;10:11. doi: 10.1186/1472-6874-10-11 (PMC2858093; doi:10.1186/1472-6874-10-11)
Supplement: Additional file 1 — Search algorithm in Medline. [file 1472-6874-10-11-S1.DOC]

| ("Uterine cervical dysplasia"[MeSH Terms] OR ("uterine"[All Fields] AND "cervical"[All Fields] AND "dysplasia"[All Fields]) OR "uterine cervical dysplasia"[All Fields] OR ("cervix"[All Fields] AND "dysplasia"[All Fields]) OR "cervix dysplasia"[All Fields]) OR ("cervical intraepithelial neoplasia"[MeSH Terms] OR ("cervical"[All Fields] AND "intraepithelial"[All Fields] AND "neoplasia"[All Fields]) OR "cervical intraepithelial neoplasia"[All Fields]) OR ("cervix uteri"[MeSH Terms] OR ("cervix"[All Fields] AND "uteri"[All Fields]) OR "cervix uteri"[All Fields]) OR ("uterine cervicitis"[MeSH Terms] OR ("uterine"[All Fields] AND "cervicitis"[All Fields]) OR "uterine cervicitis"[All Fields] OR "cervicitis"[All Fields]) OR ("uterine cervical neoplasms"[MeSH Terms] OR ("uterine"[All Fields] AND "cervical"[All Fields] AND "neoplasms"[All Fields]) OR "uterine cervical neoplasms"[All Fields] OR ("cervix"[All Fields] AND "carcinoma"[All Fields]) OR "cervix carcinoma"[All Fields]) OR (squamous[All Fields] AND intraepithelial[All Fields] AND lesion[All Fields]) OR ("uterine cervical neoplasms"[MeSH Terms] OR ("uterine"[All Fields] AND "cervical"[All Fields] AND "neoplasms"[All Fields]) OR "uterine cervical neoplasms"[All Fields]) OR (cervical[All Fields] AND lesions[All Fields]) OR (HPV[All Fields] OR (("humans"[MeSH Terms] OR "humans"[All Fields] OR "human"[All Fields]) AND ("papilloma"[MeSH Terms] OR "papilloma"[All Fields]) AND ("viruses"[MeSH Terms] OR "viruses"[All Fields] OR "virus"[All Fields])) OR (("humans"[MeSH Terms] OR "humans"[All Fields] OR "human"[All Fields]) AND ("papillomaviridae"[MeSH Terms] OR "papillomaviridae"[All Fields] OR "papillomavirus"[All Fields]))))  AND  (("cryotherapy"[MeSH Terms] OR "cryotherapy"[All Fields]) OR ("cryosurgery"[MeSH Terms] OR "cryosurgery"[All Fields])  OR  (("uterine cervical neoplasms"[MeSH Terms] OR ("uterine"[All Fields] AND "cervical"[All Fields] AND "neoplasms"[All Fields]) OR "uterine cervical neoplasms"[All Fields] OR ("cervical"[All Fields] AND "cancer"[All Fields]) OR "cervical cancer"[All Fields]) AND pretreatment[All Fields]) OR (cervical[All Fields] AND precancer[All Fields] AND ("therapy"[Subheading] OR "therapy"[All Fields] OR "treatment"[All Fields] OR "therapeutics"[MeSH Terms] OR "therapeutics"[All Fields]))  OR  LLETZ[All Fields] OR (large[All Fields] AND loop[All Fields] AND excision[All Fields] AND transformation[All Fields] AND zone[All Fields]) OR LEEP[All Fields] OR (Loop[All Fields] AND Electrosurgical[All Fields] AND Excisional[All Fields] AND ("methods"[MeSH Terms] OR "methods"[All Fields] OR "procedure"[All Fields])))  AND  (("Afghanistan OR [other low income or middle income countries by name]))) |
| --- |
