# Supplementary material for: Are treatments for cervical precancerous lesions in less-developed countries safe enough to promote scaling-up of cervical screening programs? A systematic review
Source: BMC Womens Health. 2010 Apr 1;10:11. doi: 10.1186/1472-6874-10-11 (PMC2858093; doi:10.1186/1472-6874-10-11)
Supplement: Additional file 2 — Characteristics of cryotherapy studies, and studies of cryotherapy versus LEEP, included in the review. [file 1472-6874-10-11-S2.DOC]

Additional file 2. Characteristics of cryotherapy studies, and studies of cryotherapy versus LEEP, included in the review

| Study (sample characteristics) | Management / Study groups | N | Study design | Study setting | Participants’ age (y) | Study period | Follow-up visits | Quality Score* |
| --- | --- | --- | --- | --- | --- | --- | --- | --- |
| Cryotherapy studies  *Bhatla et al, 2009* [24]  Women with biopsy-confirmed CIN | Cryotherapy | 43 | PCS | India | 25-59** | 2004-2005 | 1 mo, 13 mo | 2 |
| *Nene et al, 2008* [48] Women with positive VIA test, cytology or HPV DNA test, and colposcopically diagnosed CIN 1-3 | Biopsy + Cryotherapy | 574 | RCT | India | 30-59** | NR | 3 mo, 1 y | 2 |
| *Luciani et al, 2008* [25]  Women with positive VIA test and VIAM test. | Biopsy + Cryotherapy | 1194 | PCS | Peru | 25-49 | 2000-2004 | 30 min, 2 wk, 1 y | 2 |
| *Sankaranarayanan et al, 2007* [51]  Women with positive VIA test and colposcopically diagnosed CIN 1-3 | Biopsy + Cryotherapy | 2513 | RCT | India | 30-59** | NR | 1 y | 2 |
| *Blumenthal et al, 2007* [27]  Women with positive VIA test. | Cryotherapy | 427 | PCS | Ghana | 25-45** | 2001- 2004 | 3 mo, 1 y | 2 |
| *Mathers et al, 2005* [28]  Women with positive VIA test. | Cryotherapy | 121 | PCS | Guatemala | median,33 (13-96)** | NR | NR | 1 |
| *Denny et al, 2005* [29]  Women with positive HPV DNA test or VIA test. | Cryotherapy | 949 | RCT | South Africa | 35-65** | 2000- 2002 | 4 wk, 6 mo, 12 mo | 4 |
| *Coffey et al, 2005* [26]  Women with positive VIA test and VIAM test. | Biospy + Cryotherapy | 293 | PCS | Peru | 25-49 | Jul 2001-Oct 2001 | 5 wk (max. 13 wk) | 2 |
| *Gaffikin et al, 2003* [30]  Women with positive VIA- and VIAM tests. | Cryotherapy | 756 | PCS | Thailand | 30-45** | Feb 2000-Oct 2000 | 3 mo, 1 y | 2 |
| *Doh et al, 1999* [31]  Women with positive cytological smear test confirmed by colposcopy. | Cryotherapy | 102 | PCS | Cameroon | 20-60** | 1994-1996 | 2 wk, 6 wk, 10 wk,  6 mo, 9 mo, 12mo | 1 |
| *Adewole et al, 1998* [32]  Women with biopsy-confirmed CIN 1-3. | Cryotherapy | 23 | RCS | Nigeria | NR | 1994-NR | 9 mo to 3 y | 0 |
| Studies of cryotherapy versus LEEP |  |  |  |  |  |  |  |  |
| *Chirenje et al, 2003* [53] Women with a biopsy confirmed HSIL who accepted HIV testing (subsample of [54]). | Cryotherapy  LEEP | 75  72 | RCT | Zimbabwe | 31.9±5.7 | 1997-1998 | 2 wk, 6 mo, 12 mo | 3 |
| *Chirenje et al, 2001* [54]  Women with a biopsy confirmed HSIL | Cryotherapy  LEEP | 200  200 | RCT | Zimbabwe | 25-55 | 1997-1998 | 2 wk, 6 mo, 12 mo | 4 |

Abbreviations: LEEP=loop electrosurgical excisional procedure; N=sample size; CIN=cervical intraepithelial neoplasia; HPV= human papilloma virus; HSIL=high-grade squamous intraepithelial lesion; wk=week; mo=month; y=year; RCT=randomized control trial; PCS=prospective cohort study; RCS=retrospective case series; VIA=visual inspection with acetic acid; VIAM=magnified visual inspection with acetic acid; NR=not reported.

References [25,26] reported on different sets of cryotherapy-related harms assessed in the context of the TATI project in the Department of San Martin, Peru

References [53,54] reported on adverse outcomes of the University of Zimbabwe/JHPIEO randomized trial of cryotherapy versus LEEP (entire study sample [54]; HIV-specific results [53]).

*Quality of harm assessment based on criteria derived from Dindo’s scoring system [23].

**Age of all study participants (age of patients who underwent treatment was not provided)
