# Supplementary material for: Are treatments for cervical precancerous lesions in less-developed countries safe enough to promote scaling-up of cervical screening programs? A systematic review
Source: BMC Womens Health. 2010 Apr 1;10:11. doi: 10.1186/1472-6874-10-11 (PMC2858093; doi:10.1186/1472-6874-10-11)
Supplement: Additional file 3 — Characteristics of LEEP-only studies included in the review. [file 1472-6874-10-11-S3.DOC]

Additional file 3. Characteristics of LEEP-only studies included in the review.

| Study (sample characteristics) | Study groups | N | Study design | Study setting | Participants’ age (y) | Study period | Follow-up visits | Quality Score* |
| --- | --- | --- | --- | --- | --- | --- | --- | --- |
| *Kietpeerakool et al, 2009* [33]  Women with biopsy-proven CIN 2-3 / microinvasive lesion, persistent CIN 1, HSIL on cervical smear, or “see and treat”: approach. | HIV infected  HIV uninfected | 70  719 | PCS | Thailand | 37.5±6.6  45.8±9.1 | 2004-2008 | 2 wk, NR | 3 |
| *Ayhan A et al, 2009* [37]  Women with CIN 3 and positive ectocervical margins. | Repeat LEEP | 56 | RCS | Turkey | 24-48 | 2000-2006 | every 3 mo ( y 1)  every 6 mo (y 2-3) | 0 |
| *Sankaranarayanan et al, 2009* [49] Women with positive VIA test, cytology or HPV DNA test, and colposcopically diagnosed CIN 1-3 + contraindication to cryotherapy, or unsatisfactory colposcopy. | All participants | 1141 | RCT | India | 30-59** | 2000-2003 | 3 mo, 1 y | 2 |
| *Rema et al, 2008* [38] Women with biopsy-confirmed CIN 1-3. | All participants | 283 | PCS | India | 25-59** | 2000-2005 | 1 y | 3 |
| *Pfaendler et al, 2008* [19]  Women with positive VIA test, ineligible for cryotherapy. | HIV infected  HIV uninfected  Unknown status | 465  116  116 | PCS | Zambia | NR | 2006-2007 | 6 wk, 6 mo, 12 mo | 2 |
| *Bozanovic et al, 2007* [39]  Women with CIN 1-3. | All participants | 72 | RCS | Serbia | NR | NR | NR | 0 |
| *Kietpeerakool et al, 2007* [34]  Women with HSIL on Pap smear or on colposcopic biopsy, unsatisfactory colposcopy, margin involvement of previous LEEP, or “other” indication. | Repeat LEEP  First LEEP | 78  472 | PCS | Thailand | Mean, 47.5  NR | 2004-2007 | 24 h^†^, 2 wk, 4 wk^†^ | 2 |
| *Kietpeerakool et al, 2006* [35]  Women with HSIL on Pap smear or colposcopic biopsy, unsatisfactory colposcopy, margin involvement of previous LEEP, or “other” indications. | All participants | 226^††^ | PCS | Thailand | 26-72 | 2004-2005 | 24 h^†^, 2 wk, 4 wk^†^ | 2 |
| *Kietpeerakool et al, 2006* [36]  Women with unsuspected invasive cervical cancer. | Micro-invasive cervical cancer | 46 | PCS | Thailand | 45.6±9.8 | 2004-2005 | 24 h^†^, 2 wk, 4 wk^†^ | 2 |
| *Kietpeerakool et al, 2006* [40]  HIV-infected and HIV-uninfected women with abnormal Pap smear and colposcopic impression. | HIV infected  HIV uninfected | 60  60 | RCS | Thailand | 35.9±8.7  40.1±6.5 | 1998-2004 | 2 wk, 6 mo, 1 y | 2 |
| *Sankaranarayanan et al, 2004* [52]  Women with positive VIA test and colposcopically diagnosed CIN 1-3. | All participants | 148 | RCT | India | 30-59 | 2000-2003 | 1 y | 2 |
| *Suntornlimsiri et al, 2004* [41]  Women with HSIL on Pap smear and abnormal colposcopic impression. | All participants | 178 | RCS | Thailand | 28-71 | 2003-2004 | “Weekly” | 1 |
| *Wozniak et al*, 2003 [42] Women with cervical dysplasia. | All participants | 28 | RCS | Poland | NR | NR | 8 wk | 0 |
| *Kattukaran et al, 2002* [43]  Women with LSIL or HSIL upon colposcopy. | All participants | 18 | PCS | India | NR | 1999-2000 | 7-10 d^†^ | 3 |
| *Huang et al, 1999* [44]  Women who underwent LEEP or cold knife conization within 6 months prior to having a hysterectomy. | All participants | 73 | RCS | China | 46.3±10.3 | 1992-1997 | Unclear (max. 6 mo) | 1 |
| *Darwish et al, 1998* [45] Women with abnormal colposcopic findings. | All participants | 66 | PCS | Egypt | NR | 1995-1997 | 3 mo, 6 mo, 12mo | 0 |
| *Ghosh et al, 1997* [46] Women with diagnosis of HSIL or persistent LSIL confirmed by colposcopy. | All participants | 660 | PCS | Honduras | Mean, 34.2 | 1993-1995 | 2 wk, 3 mo, then every 3-6 mo | 2 |
| *Santos et al, 1996* [47]  Women with cytological and colposcopic evidence of CIN. | All participants | 149 | RCT | Peru | NR | 1992-1994 | 7 d and 1, 3, 6, 9,  12, 16, 20, 24 mo | 3 |

Abbreviations: see Table 1.

References [33-36, and 40] reported on outcomes of interest in different subgroups of two series of women treated at Chiang Mai University Hospital, Thailand.

References [48 (Table 1) and 49] reported on outcomes of cryotherapy and LEEP in a screening intervention trial conducted in Osmanabad District, Maharashtra State, India. Reference [50] reported one additional complication among participants to the same study (hysterectomy for uncontrollable bleeding after LEEP).

References [51 (Table 1) and 52] reported on outcomes of cryotherapy and LEEP in a screening intervention trial conducted in Dindigul District, Tamil Nadu State, India.

*Quality of harm assessment based on criteria derived from Dindo’s scoring system [23].

**Age of all study participants (age of patients who underwent treatment was not provided)

^†^Phone call

^††^226 LEEP performed in 206 women
