# Supplementary material for: Are treatments for cervical precancerous lesions in less-developed countries safe enough to promote scaling-up of cervical screening programs? A systematic review
Source: BMC Womens Health. 2010 Apr 1;10:11. doi: 10.1186/1472-6874-10-11 (PMC2858093; doi:10.1186/1472-6874-10-11)
Supplement: Additional file 4 — Summary of evidence on safety of cryotherapy and LEEP for CIN in less-developed countries, 1995-2009. [file 1472-6874-10-11-S4.DOC]

**Additional file 4. Summary of evidence on safety of cryotherapy and LEEP for CIN in less-developed countries, 1995-2009**

|  | **Cryotherapy** | | **LEEP** | |
| --- | --- | --- | --- | --- |
| **Harm** | **Patients***  Median % (Range %) | **Studies****  N (Sample size) | **Patients***  Median % (Range %) | **Studies^**^**  N (Sample size) |
| Pain   - During or immediately after procedure   - Mild, moderate   - Severe; required analgesics - Longer lasting or associated with menses | 29.2 (1.0-40.2)  3.6 (1.0-6.2)  9.0 (0.1-43.5) | 6 (665; 200-1026)  2 (478; 200-756)  8 (387; 23-1026) | 3.5 (2.5-35.5)  0.7 (0.4-1.0)  66.8 (46.2-87.4) | 3 (283; 200-1141)  2 (242; 200-283)  2 (213; 200-226) |
| Vaginal discharge   - Watery, annoyance, or unspecified - Offensive, disconcerting | 65.0 (8.7-92.4)  2.0 (0.9-68.2) | 5 (200; 23-949)  7 (293; 43-1026) | “most” (78.5-100)  3.2 (0.5-79.0) | 3 (200; 18-226)  3 (283; 200-1141) |
| Bleeding   - Minimal, slight, uncomplicated - Intra-operative   - Excessive, or controlled by vaginal packing or suture - Immediate / Primary / Early   - Persistent, uncomplicated   - No hospitalization; controlled with Monsel’s solution/electrocautery - Delayed / Secondary / Late   - Blood clots; no hospitalization; controlled with Monsel’s solution/electrocautery - Severe bleeding (immediate or delayed)   - Hospitalization, transfusion, suturing   - Hysterectomy | **1.3** (0.7-1.9)  **1.0** (0.5-1.5)  **4.7** (2.0-40.0)  **0.5**  **0.0**  **0.0** (0.0-0.1) | 3 (574; 427-1026)  2 (478; 200-756)  5 (756; 43-1194)  1 (627)  1 (1194)  6 (665;43-1026) | **2.8 - 5.8** days^†^  **1.2** (0.0-2.3)  **2.3** (1.6-7.9^††^)  **0.0** (0.0-2.0)  **7.9** (4.2-14.6^‡^)  **0.5** (0.3-3.3)  **4.5** (2.5-79.0)  **3.3** (1.5-5.2)    **0.4** (0.0-0.8)  **0.0** (0.0-0.14^‡‡^) | 1. (469; 149-789)   2 (404; 18-789)  3 (226;120-748)  3 (178; 73-200)  3 (78; 46-789)  3 (748; 120-789)    4 (175; 18-283)  7 (149; 56-1141)  4 (516; 148-789)  5 (283; 18-1141) |
| Infection   - Fever; cervical tenderness; infection requiring antibiotherapy - Pelvic inflammatory disease | **2.0** (0.0-2.6)  **0.0** (0.0-0.0) | 5 (574; 102-1026)  5 (574; 43-1026) | **1.2** (0.0-10.0)  **0.0** (0.0-0.7) | 10 (143 ; 72-1141)  5 (149; 73-1141) |
| Related to local anesthesia   - Trembling of lower extremities; no treatment required - Hypertension; no treatment required |  |  | **0.3**  **26.0** | 1. (748)   1 (149) |
| Reproductive-related   - Functional cervical stenosis - Cervical incompetence | **0.0** (0.0-0.0)  **0.0** | 1. (387; 23-1026)   1 (102) | **0.0** (0.0-8.0) | 8 (242; 72-1141) |
| Others   - Anaphylactic reaction - New troubling symptom, or consulted clinician at 1-month visit - Unscheduled visit for problem - Visit for problem requiring outpatient treatment - Hospital admission/unintended surgery - Accidental freezing or burn of vaginal wall - Major complication | **0.0** (0.0-0.0)  **25.2**  **5.5** (4.4-9.7)  **1.1** (0.0-2.2)    **0.0** (0.0-0.2)  **0.2** (0.1-0.8)  **0.0** (0.0-0.0) | 1. (1026; 43-1194) 2. (949)   3 (756; 427-949)  2 (592; 427-756)  5 (756; 43-1026)  3 (574; 121-1026)  3 (756; 427-1026) | **5.6**    **2.4** (0.4-4.4)  **0.0** (0.0-0.0) | 1 (18)   1. (712; 283-1141)   2 (83; 18-148) |

*Percentage of patients who experienced harm (median and range)

**Studies reporting on harm: N = number of studies; “Sample size” = median and range of sample size

^†^Mean number of days bleeding (range across studies). When reported, standard deviation varied from 2.6 to 4.7 days ([33], repeated LEEP [35]).

^††^The high value of 7.9 percent was presented in an early report [35] of study [33].

^‡^The high value of 14.6 percent was estimated among 78 patients of study [33] who had repeated LEEP [34]

^‡‡^Assuming that 5 percent of LEEP in study [44] resulted in hysterectomy for treatment failure.

Note: one estimate of pain during or immediately after cryotherapy (4.4% [32]) and two estimates of “bleeding” (one after cryotherapy, 0% [32] and one after LEEP, 2.8% [39]) were excluded from Table 4 because no indication of timing-severity was provided.
